# A Light-Up Probe for Detection of Adenosine in Urine Samples by a Combination of an AIE Molecule and an Aptamer

**DOI:** 10.3390/s17102246

**Published:** 2017-09-29

**Authors:** Yingying Hu, Jingjing Liu, Xiangyu You, Can Wang, Zhen Li, Weihong Xie

**Affiliations:** 1 Department of Food and Pharmaceutical Engineering, Key Laboratory of Fermentation Engineering (Ministry of Education), Hubei University of Technology, Wuhan 430068, China; 15518581008@163.com (Y.H.); jliu3971@gmail.com (J.L.); limnamil@gmail.com (X.Y.); 2 Department of Chemistry, Wuhan University, Wuhan 430072, China; canwang-scola@whu.edu.cn (C.W.); lizhen@whu.edu.cn (Z.L.)

**Keywords:** aggregation-induced emission, aptamer, adenosine, 1,2-bis[4-(triethylammoniomethyl)phenyl]-1,2-diphenylethenedibromide (TPE-2N+)

## Abstract

A light-up fluorescent probe for the detection of adenosine was constructed with an AIE (aggregation-induced emission) molecule and a DNA aptamer. The AIE molecule was used as a signal generator, and the DNA aptamer was used as a recognition element for adenosine. The emission of the AIE molecule was due to its intramolecular rotation restriction induced by the aptamer upon binding of adenosine. The optimal component ratio of the probe was AIE molecule/DNA aptamer = 100 (μM/μM). The calibration curve of adenosine detection showed a linear range of 10 pM to 0.5 μM with an R^2^ of 0.996, and the detection limit of the probe was 10 pM. The probe exhibited a good selectivity to adenosine against its analogs (uridine, guanosine, and cytidine). The probe was used to detect adenosine in urine samples, a recovery from 86.8% to 90.0% for the spiked concentrations of adenosine (0.01, 0.05, 0.1 μM). The relative standard deviation from 1.2% to 2.0% was obtained. The intra-day and inter-day tests also showed good precisions, with measurement RSD values of 2.3% and 2.1%, respectively.

## 1. Introduction

Aggregation-induced emission (AIE) [1] is a phenomenon that a luminescent molecule is non-emissive when it is dissolved in a solution. However, it becomes emissive when it is in an aggregation state. The unique photo physical phenomenon was first discovered by Tang and his colleagues in 2001. They later explained that the unique phenomenon was caused by a restriction of intramolecular rotation (RIR) of AIE molecules. Light-up probes are more preferred than light-off probes because they give less false-positive responses, so that interests are drawn to design and synthesis of AIE probes for bio-detection purposes [2,3,4,5,6,7]. In principle, if an analysis target can induce the restriction of intramolecular rotation of an AIE molecule, it will light up the fluorogen: the AIE molecule will give a fluorescent response to the analysis target. To restrict the intramolecular rotation of an AIE molecule, an interaction between the AIE molecule and the target molecule is usually required. Therefore, elements with high affinity for the analysis target must be provided to AIE probes. However, having a high affinity is not enough. AIE probes must also have specificity for their targets. In order to improve the specificity of AIE probes, AIE molecules of different structures have been designed and synthesized to conjugate recognition functional groups or associate with recognition elements to improve the affinity and selectivity of the probes. In this work, we used an adenosine-specific aptamer as the recognition element for the target molecule, adenosine.

Aptamers [8] are single-stranded oligonucleotides that have specific recognition function for peptides, proteins, and small organic molecules. The specific recognition function of aptamers for target molecules is based on their unique sequences and three-dimensional folded structures. Aptamers can undergo significant conformational changes into hairpins, stem-loops, or G-quadruplexs after they bind to their targets. The Aptamer that can specifically bind adenosine has been found. It is a G-rich oligonucleotide and can form G-quartets structures in the presence of adenosine [9,10].

Adenosine (A) [11] is an endogenous nucleoside that plays important roles in many biochemical processes, such as energy transfer by forming molecules like adenosine triphosphate (ATP) and adenosine diphosphate (ADP) and signal transduction by forming signally molecules like cyclic adenosine monophosphate (cAMP). It is also a neuromodulator that plays roles of promotion of sleep and suppression of arousal. In addition, adenosine regulates the blood flow to various organs through vasodilation. In recent years, it was found that adenosine’s concentration in the extracellular tissue surrounding tumors was higher than that under healthy conditions. This phenomenon was due to the hypoxic microenvironment of tumors that trigger a strong inflammatory response. Therefore, adenosine is a possible biomarker for cancer [12] and may be used for monitoring progress of diseases.

In this work, a fluoresce probe by the combination of an AIE molecule: 1,2-bis[4-(triethylammoniomethyl)phenyl]-1,2-diphenylethenedibromide (TPE-2N+) and an adenosine-specific aptamer (ABA) for the determination of adenosine in urine samples was described. When adenosine was present, the aptamer bound to the target molecule and formed a G-quadruplex that could bind and aggregate the TPE-2N+. The AIE molecule thus lighted up and gave response to adenosine.

## 2. Materials and Methods

### 2.1. Materials

Adenosine (A), cytidine (C), uridine (U), and guanosine (G) were purchased from Shanghai source leaf Biological Technology Co., Ltd. (Shanghai, China). 1,2-bis[4-(triethylammoniomethyl)phenyl]-1,2-diphenylethenedibromide (TPE-2N+) were obtained from the chemistry department, Wuhan University. Three hydroxymethyl aminomethane (Tris) and HCl were bought from Sinopharm Chemical Reagent Co., Ltd. (Shanghai, China). The oligonucleotides were synthesized by Sangon Biotechnology Co. Ltd. (Shanghai, China) with the following sequences:

Adenosine aptamer (ABA): 5′-ACCTGGGGGAGTATTGCGGAGGAAGGT-3′;

All chemicals used were analytical grade, and the ultrapure water was deionized to 18.25 MΩ·cm in a water purification system from Angel Electric Appliance Co., Ltd. (Wuxi, China).

### 2.2. Measurements

An LS-55 fluorescence spectrometer of PerkinElmer (Shanghai, China) was used to record the fluorescence spectra. The emission spectra were recorded in the range from 350 to 600 nm with both excitation and emission slits of 10 nm. All fluorescence detections were carried out under room temperature.

### 2.3. The Emission Behavior of (TPE-2N+) in Tris Buffer

The emission of (TPE-2N+) in Tris buffer was measured under an excitation wavelength of 400 nm, the dependence of the fluorescence intensity at the maximum emission wavelength on the concentration of the fluorogen was investigated within a concentration range of 5–100 μM. The variation of TPE-2N+ fluorescence intensity over a time period of 30 min was detected under two concentrations of 5 and 50 μM. The recipe of the (TPE-2N+) solutions is provided in Table S1.

### 2.4. The Viability of (TPE-2N+) + ABA Probe

To verify the feasibility of the proposed probe, the fluorescence intensity of the following systems was measured:

(1) 10 mM Tris-HCl;
(2) 10 mM Tris-HCl + 10 μM (TPE-2N+);
(3) 10 mM Tris-HCl + 10 μM (TPE-2N+) + 0.1 μM ABA;
(4) 10 mM Tris-HCl + 10 μM (TPE-2N+) + 0.1 μM A;
(5) 10 mM Tris-HCl + 10 μM (TPE-2N+) + 0.1 μM ABA + 0.1 μM A.

The samples were prepared according to the recipe in Table S2. The stock solutions were 100 μM (TPE-2N+), 100 μM ABA, and 1 μM adenosine and 10 mM pH 7.4 Tris-HCl up to 200 μL.

### 2.5. Optimization of the Concentration and the Concentration Ratio of the (TPE-2N+) and the ABA

The component of the probe was optimized by varying the concentrations of (TPE-2N+) and ABA, and their ratio. (TPE-2N+) solutions of 5 μM, 10 μM, and 20 μM and ABA of 0.1 μM, and 0.3 μM, 0.5 μM were prepared by diluting the stock 100 μM (TPE-2N+) and 100 μM ABA with Tris-HCl buffer, respectively. The concentration ratio of (TPE-2N+): ABA (μM:μM) was adjusted to 5:0.5, 10:0.1, 10:0.3, 10:0.5, and 20:0.5. Adenosine solutions of 0, 0.01, 0.05, 0.1, 0.2, and 0.5 μM were prepared by diluting a 1μM adenosine solution into the detection systems. The fluorescence intensity of the systems was then measured under the same conditions as described in the above sections.

### 2.6. The Calibration Curve of Adenosine Detection

Solutions of adenosine were prepared with a final concentration of 0, 1 × 10^−5^, 5 × 10^−5^, 1 × 10^−4^, 5 × 10^−4^, 1 × 10^−3^, 5 × 10^−3^, 1 × 10^−2^, 5 × 10^−2^, and 1 × 10^−1^ μM. (TPE-2N+) and ABA were added into the adenosine solutions with the optimal ratio with a concentration of 10 μM and 0.1 μM, respectively. The fluorescence intensity of the systems was then measured.

### 2.7. Specificity of (TPE-2N+)-ABA Detection System

The specificity of the probe for adenosine was examined using cytidine, uridine, guanosine, and the mixture of the nucleotides as the controls. The nucleotide samples were prepared according to the recipe in Table S3. The stock solutions were 100 μM of TPE, 100 μM of ABA, 1 μM of nucleotides, and 10 mM pH7.4 Tris-HCl.

### 2.8. Analytical Application to Urine Samples

To investigate the practical application of the probe in more complicated conditions, the detection of adenosine was carried out in human urine samples. A healthy urine was used as the blank sample; adenosine was added to prepare the spiked samples. The spiking concentrations were 0.01, 0.05, and 0.1 μM. The sample solutions were diluted 100 times before the detection. A working curve was also made using the healthy urine, and a linear regression equation for adenosine was obtained from a calibration curve using concentrations from 0 to 0.1 μM. A number of detections were carried out to investigate the precision and recovery of the probe.

## 3. Results and Discussions

### 3.1. The Detection Principle

**Scheme 1:**
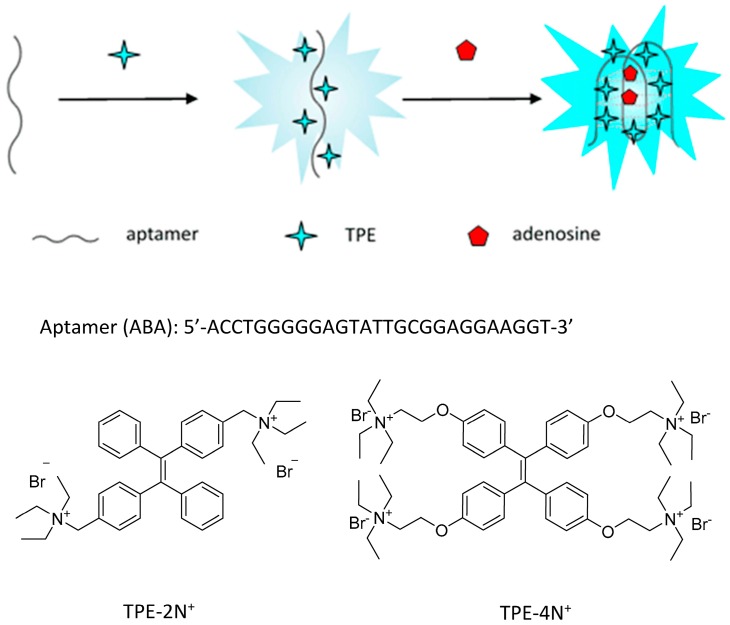
Analytical principle of the tetraphenylethene (TPE)-aptamer probe for adenosine detection.

(TPE-2N+) is an amino functionalized derivative of tetraphenylethene (TPE). With the amino functional groups, the solubility of (TPE-2N+) in an aqueous solution is improved so that it can be applied as a probe for bio-samples. Being an AIE molecule, (TPE-2N+) shares the common (AIE) characteristics of aggregation-induced emission. Therefore, any process or molecules that cause the restriction of intramolecular rotation of (TPE-2N+) will light it up. It has been reported that 1,1,2,2-tetrakis[4-(2-triethylammonioethoxy)phenyl]ethene tetrabromide (TPE-4N+), one of (TPE-2N+)’s structure similar, could bind to a guanine-rich DNA strand and was highly affinitive to the G-quadruplex structure of the DNA strand. The high affinity of the TPE-4N+ to G-quadruplex was associated with a geometric fit aided by an electrostatic attraction. Upon the electrostatic attraction, the TPE’s intramolecular rotation was restricted and its emission was turned on. A K^+^ biosensor was developed using TPE-4N+ because it was specific to the K^+^-induced and -stabilized G-quadruplex [13]. Similarly, the adenosine-specific aptamer (ABA) used in this work is also a G-rich repeat sequences. It was evidenced that in the presence of adenosine, ABA bound adenosine and performed a conformational change to a G-quadruplex [9,10]. Several aptasensors based on this conformational change had been seen reported [14,15,16,17,18]. However, the combination of ABA with an AIE fluorogen has not been reported. The sensing principle is shown in Scheme 1. In the absence of adenosine, ABA takes a random conformation, and its negatively charged phosphate groups may attract the positive TPE-2N+ via an electrostatic interaction. Upon the electrostatic attraction, at least one rotation of the benzene groups of TPE-2N+ is restricted and emission of the TPE-2N+ is turned on. However, the conformation of ABA is random in the absence of adenosine, so that the ABA-TPE interaction is random and the emission is random. In the presence of adenosine, the aptamer will undergo a conformational change to G-quadruplex. As was reported in previous works, TPE amino functionalized derivatives had strong affinity for G-quadruplex structures. The strong affinity was due to the geometric fit between the TPE molecule and the DNA G-quadruplex, which was aided by the electrostatic attraction between amino groups of the TPE molecule and the DNA phosphate groups and a hydrophobic interaction between the aromatic core of the TPE and the deoxyribose regions of the DNA [19]. The intramolecular rotation of the TPE molecule was strongly restricted and a large enhance in emission was observed. Since the formation of ABA G-quadruplex is specifically induced by adenosine, TPE-2N+ can be used as a bio-probe to detect adenosine.

### 3.2. The Emission Behavior of TPE Fluorogen in Tris Buffer

**Figure 1:**
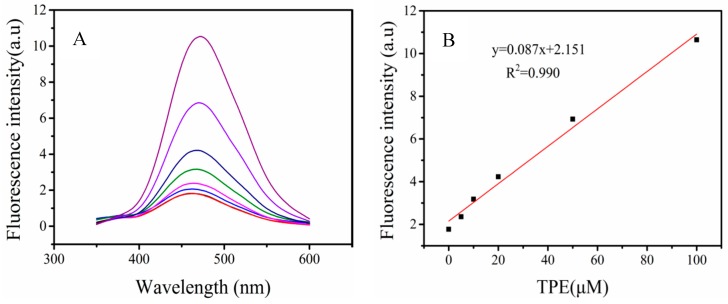
(**A**) Fluorescence emission spectra of different concentrations of TPE-2N+ in Tris-HCL buffer. From bottom to top: 0, 5, 10, 20, 50, 100 μM. (**B**) The linear relationship between the fluorescence intensity and the concentration of TPE-2N+ in Tris-HCL buffer.

**Figure 2:**
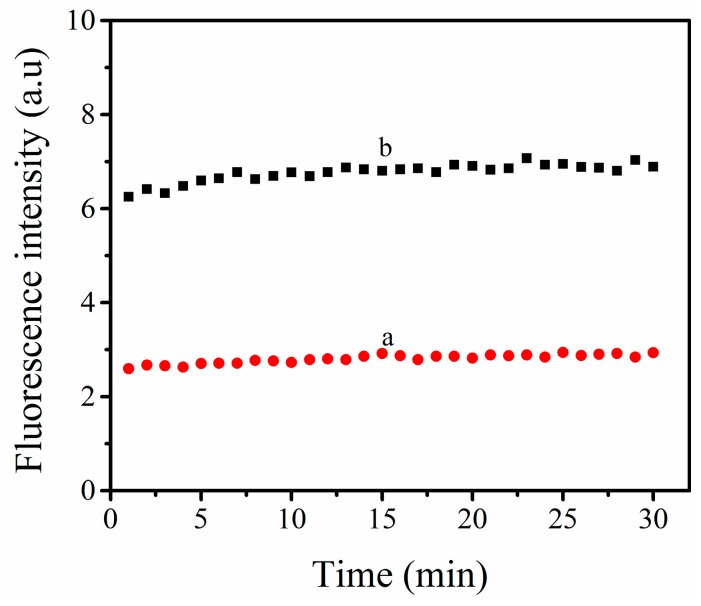
Fluorescence intensity of a: 5 μM and b: 50 μM of TPE-2N+ in Tris-HCL buffer over a time period of 30 min.

The fluorescence intensity of TPE-2N+ in Tris-HCl buffer was measured, the result indicated that under an excitation of 400 nm, the fluorogen gave an emission at 470 nm. The emission intensity was increased gradually with the increase of the fluorogen’s concentration (Figure 1A), and a linear relationship was obtained from 0 μM to 100 μM (Figure 1B). The stability of TPE fluorescence in Tris-HCl buffer was also examined, and a variation of TPE fluorescence intensity was measured over a time-period of 30 min. As shown in Figure 2, there is no obvious change in fluorescence intensity for the selected concentrations 5 μM and 50 μM of TPE-2N+ which suggested the TPE fluorogen had a favorable stability in Tris-HCl buffer.

### 3.3. The Viability of (TPE-2N+) + ABA Probe

**Figure 3:**
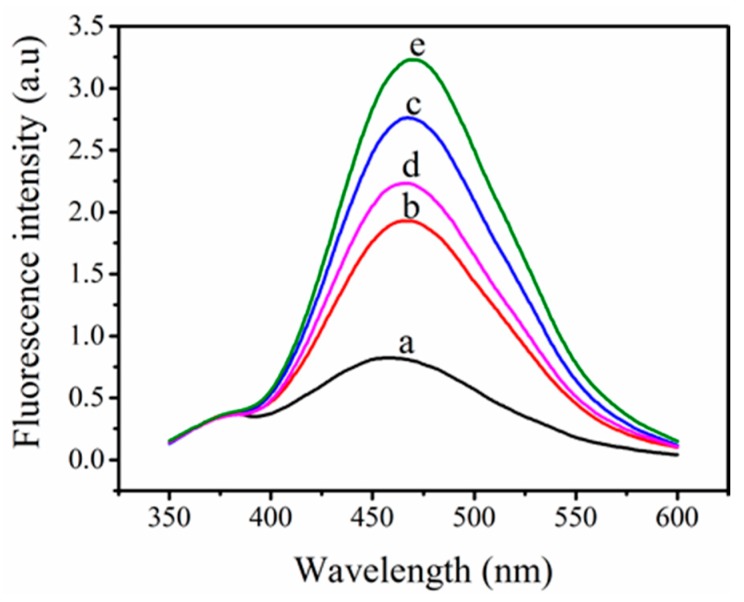
Fluorescence response of the system under different conditions. a: 10 mM Tris-HCL; b: 10 mM Tris-HCL + 10 μM TPE-2N+; c: 10 mM Tris-HCL + 10 μM TPE-2N+ + 0.1 μM ABA; d: 10 mM Tris-HCL + 10 μM TPE-2N+ + 0.1 μM Ade; e: 10 mM Tris-HCL + 10 μM TPE-2N+ + 0.1 μM ABA + 0.1 μM Ade.

To verify the feasibility of the present approach, fluorescence intensity of solutions with different components was measured. The fluorescence spectrum of these solutions are presented in Figure 3. Tris buffer exhibited a very low fluorescence intensity (Figure 3, spectrum a). Upon addition of TPE-2N+, fluorescence intensity of the system increased (Figure 3, spectrum b). Further addition of the aptamer ABA, fluorescent intensity of the system is seen to be enhanced. We attributed the enhancement to the restriction of intramolecular rotation of TPE-2N+ caused by the electrostatic attraction of the negative phosphate groups on the DNA strand (Figur 3, spectrum c). In the absence of the aptamer, TPE-2N+ gave only a small fluorescence response to adenosine that only a small increase in fluorescence intensity upon the addition of the target molecule is observed (Figure 3, spectrum d). As there was no specific interaction between (TPE-2N+) and adenosine, the presence of the target molecule did not cause strong restriction of intramolecular rotation of the fluorogen thus did not cause strong fluorescence response. When the adenosine-specific ABA was added, the aptamer would bind to the target and undergo a secondary conformational change. The G-quadruplex structure of ABA induced by adenosine could attract and restrict the intramolecular rotation of the fluorogen so that the fluorogen become highly emissive (Figure 3, spectrum e). Since the fluorescence response was generated based on the specific recognition between the aptamer and adenosine, the system of TPE-2N+/ABA could be developed as a biopeobe for adenosine sensing.

### 3.4. Optimization of the Optimal Ratio of TPE-2N+ and ABA Concentration

**Figure 4:**
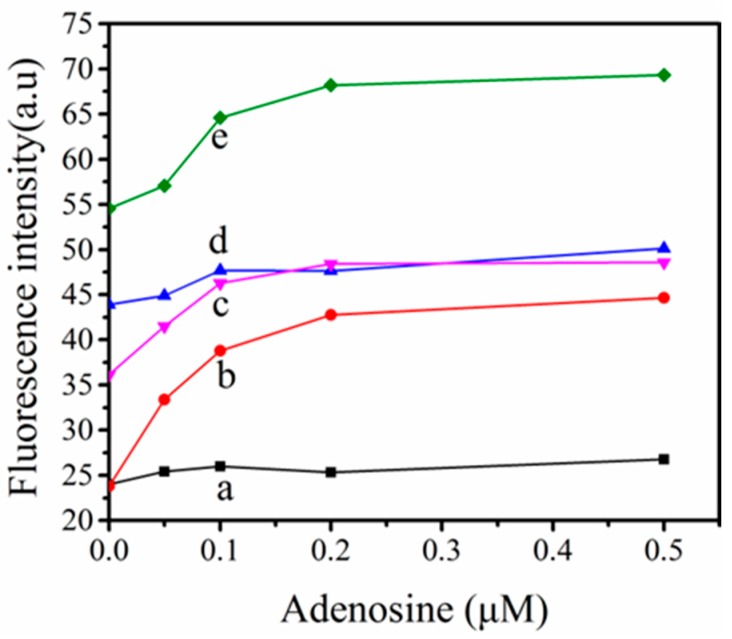
The fluorescent response toward the concentration of adenosine (0, 0.01, 0.05, 0.1, 0.2, and 0.5 μM) under different ratio of TPE-2N+ and ABA concentration, a: 5:0.5; b: 10:0.1; c: 10:0.3; d: 10:0.5, and e: 20:0.5.

In order to get the best response signals, concentrations of TPE-2N+ and ABA and their ratio in the detect system were optimized. A series of concentrations of 5 μM, 10 μM, and 20 μM and 0.1 μM, 0.3 μM, and 0.5 μM, respectively, for TPE-2N+ and ABA were tested. Plots of Figure 4a,d,e show the fluorescent response to adenosine under fixed concentration of ABA (0.5 μM) and vary concentrations (5 μM, 10 μM, and 20 μM) of TPE-2N+, respectively. Under condition a, the detection system does not show an adenosine-dependent fluorescence response; in cases of d and e, the fluorescence signal show an adenosine concentration dependence within 0–0.2 μM. Although the fluorescence response is higher with a higher TPE-2N+ concentration, the background is also higher, so that concentration of 10 μM was chosen for TPE-2N+ for the later experiments. At fixed concentration of TPE-2N+ (10 μM), the fluorescence responses under 0.1, 0.3, and 0.5 μM of ABA were measured and the results are shown respectively in Figure 3b–d. As can be seen, the fluorescence response of the detection system increase with the increase of ABA’s concentration. However, the background value also increase, considering the best signal-to-noise level, condition of b is chosen.

The concentration ratio (TPE-2N+): ABA (μM:μM) of the detection system was also optimized. The ratio of (TPE-2N+): ABA as 5:0.5, 10:0.1, 10:0.3, 10:0.5, and 20:0.5 were investigated. As shown in Figure 2, in cases of 5:0.5 and 10:0.5, the fluorescence response shows little dependence on the concentrations of adenosine (Figure 4). The adenosine concentration dependence of fluorescence response was observed under (TPE-2N+): ABA ratio of 10:0.1, 10:0.3 and 20:0.5 (Figure 4, curve b, c and e), the fluorescence intensity of the systems increase greatly within the adenosine concentration range of 0–0.2 μM. Although the fluorescence intensity is higher under (TPE-2N+): ABA ratio of 10:0.3 and 20:0.5 (Figure 4, curve c and e), the background value was much greater than b. Hence 10:0.1 was chosen as the optimal condition for the probe.

### 3.5. The Sensitivity of the Probe

**Figure 5:**
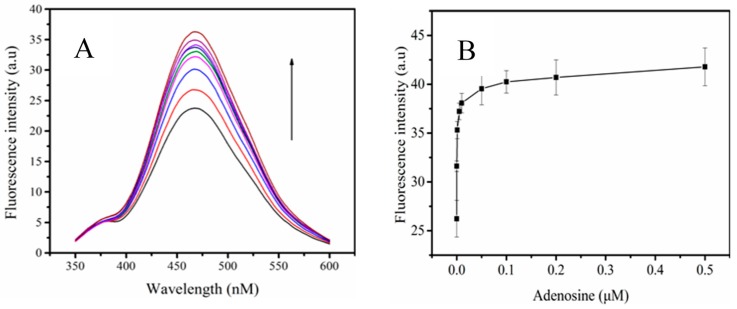

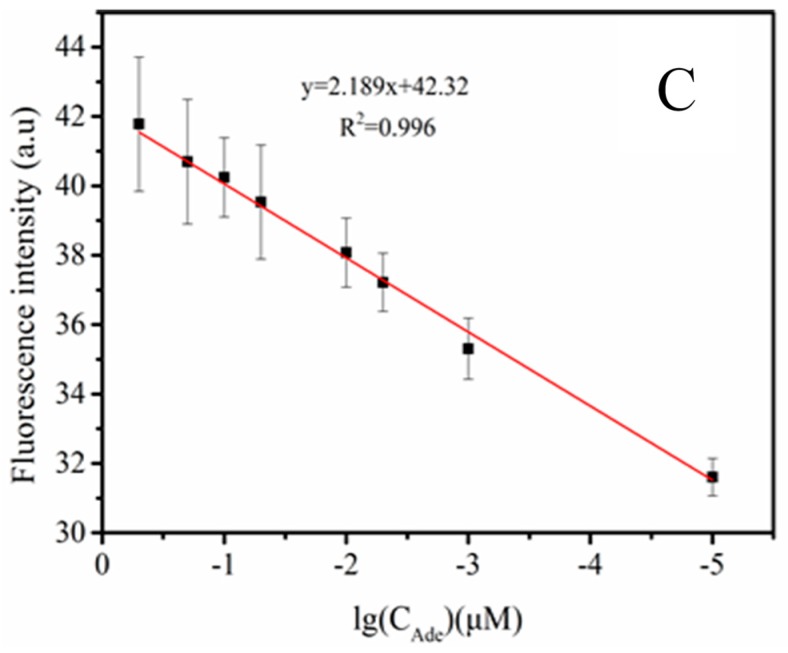
(**A**) Fluorescence emission spectra of the sensing system in the presence of different concentrations of adenosine. From bottom to top: 0, 0.00001, 0.00005, 0.0001, 0.001, 0.005, 0.01, 0.05, and 0.1 μM. (**B**) The relationship between fluorescence intensity and the concentration of adenosine. (**C**) A linearity curve (R2 = 0.996) was confirmed as the logarithm of adenosine concentration changed from 10 pM to 0.5 μM. The error bars are standard deviations of three repetitive measurements.

The ability of this fluorescence aptasensor for the quantitative detection of adenosine was studied under the optimized conditions. Figure 5A shows the different fluorescence responses to adenosine of various concentrations. As can be seen from Figure 5B, the fluorescence response increased dramatically when the concentration of adenosine increases from 0 to 0.1 μM. A linear relationship between the fluorescence response and the concentration of adenosine is obtained according to the equation of Y = 42.32 + 2.189X, where Y is the relative fluorescence intensity, and X is the logarithm of adenosine concentration Figure 5C. The linear concentration range is 10 pM to 0.5 μM with an R^2^ of 0.996. So that the detection limit of the aptasensor for adenosine can be 10 pM.

### 3.6. Specificity of TPE-ABA Detection System

**Figure 6:**
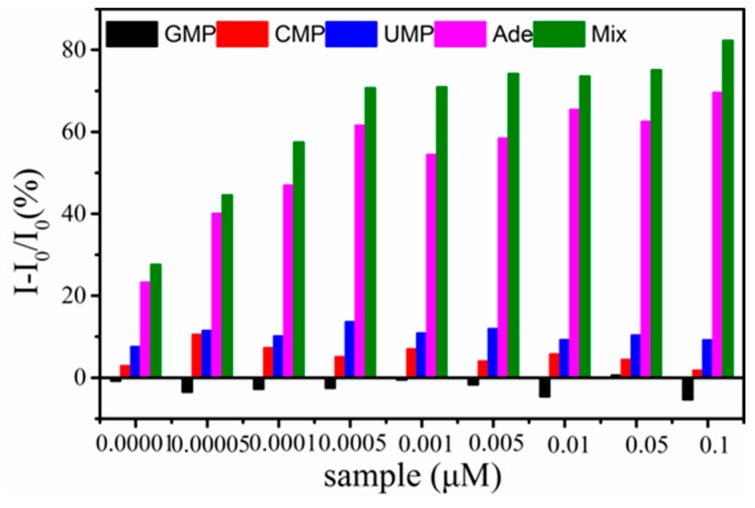
The fluorescent response of the proposed aptasensor towards adenosine and the three analogues: guanosine, uridine and cytidine, and the mixture of adenosine with the analogues. The concentration of all samples were 0.00001, 0.00005, 0.0001, 0.0005, 0.001, 0.005, 0.01, 0.05, and 0.1 μM.

The selectivity of the probe toward adenosine was examined by measuring the fluorescence responses of several adenosine analogs (uridine, guanosine, and cytidine) under the same experimental conditions. As shown in Figure 6, the TPE-2N+/ABA probe only gives a small fluorescence response to the adenosine analogs and does not show a concentration dependence of the adenosine analogs. However, it gives distinct fluorescence response to adenosine, and the fluorescence response increases with the increase of the concentration of adenosine. This result suggested that uridine, guanosine, and cytidine did not induce a conformational change of the ABA strand, so that the intramolecular rotation of TPE-2N+ was not restricted, and the emission was not turned on. The fluorescence response of TPE-2N+/ABA to adenosine in the presence of cytidine, guanosine and uridine does not show much affect by the adenosine analogs. This result indicated that the TPE-2N+/ABA probe had a sufficient specificity to adenosine against other adenosine analogs.

### 3.7. Analytical Application to Urine Samples

**Figure 7:**
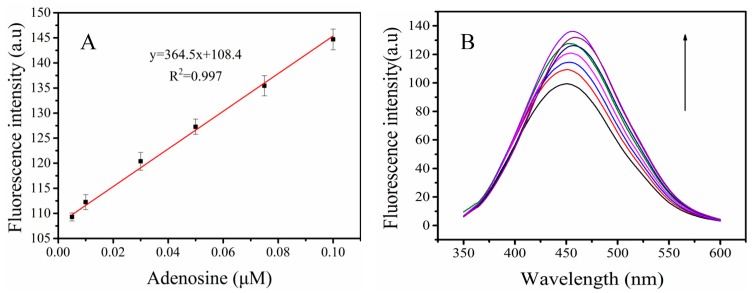
(**A**) The linear relationship between the change of fluorescence intensity and the concentration of adenosine in the urine. (**B**) shows the fluorescence spectra of ABA+TPE in urine diluted with Tris-HCL buffer after put in 0, 0.005, 0.01, 0.03, 0.05, 0.075, 0.1, and 0.12 μM of adenosine with an excitation wavelength of 470 nm.

To demonstrate the applicability of the proposed probe in the detection of adenosine in real samples, the recoveries of adenosine in human urine samples by the probe was examined. A working curve was firstly plotted choosing healthy urine as a blank. Adenosine was then spiked to the blank urine and was 100-fold diluted with Tris-HCl buffer to get final concentrations of 0.005, 0.01, 0.03, 0.05, 0.08, and 0.1 μM. A linear relationship between fluorescence intensity and the concentration of adenosine (0.05–0.1 μM) was obtained (Figure 7). Urine samples with three spiked concentrations (0.01, 0.05, and 0.1 μM) of adenosine were measured, and the measurements were quantified by using the working curve. As listed in Table 1, the obtained recoveries of the adenosine concentrations were from 86.8% to 90.0% with the relative standard deviation from 1.2% to 2.0%. The intra-day precision and inter-day precision tests indicated that the RSD values were within 2.3% and 2.1% respectively (Tables S4 and S5). The precision and recovery of this proposed method applied in complex biological samples are satisfactory. Although K+, a physiological component, was well known to stabilize G-quadruplex structure, it has been demonstrated by previous works [9,16] that K+ had little effect on the recognition and binding of the adenosine-specific aptamer to adenosine, in another word, it is not competitive to adenosine to bind the aptamer, so that the sensing based on the aptasensor was less affected by this cation even at a much higher concentration.

## 4. Conclusions

A light-up fluorescent probe was developed by the combination of an AIE molecule and a DNA aptamer for the detection of adenosine. The probe device was simple compared with other aptamer-based probes in that the detection did not involve an enzyme strategy. Besides, the DNA strand was label free, and no fluorescent compound was required to conjugate on the DNA strand. This reduced the complications of the work and the expense on the DNA strand. On the other hand, the AIE molecule was also not required to function with a recognition group. The probe showed a lower detection limit when compared with other reported data. The good recovery results and precision of the probe for the urine samples suggested that the device might be further developed into test kit for clinic applications.

## Figures and Tables

**Table 1:** Determination of adenosine in urine samples using the proposed probe.

Samples	Adenosine Spiked (μM)	Adenosine Found (μM)	Recovery (%)	RSD (%, n = 6)
1	0.01	0.0090	90.0	1.9
2	0.05	0.0434	86.8	1.2
3	0.1	0.0892	89.2	2.0

## Supplementary Materials

The following are available online at www.mdpi.com/1424-8220/17/10/2246/s1, Table S1: The recipe of different concentrations of the TPE-2N+ solutions for the examination of the emission behavior of TPE fluorogen in Tris buffer; Table S2: The recipe for investigation of the viability of (TPE-2N+) + ABA probe; Table S3: The recipe of different concentrations of nucleotide samples for the specificity experiment; Table S4: The intra-day precision of the detection by the probe for urine samples; Table S5: The inter-day precision of the detection by the probe for urine samples.
