# Supplementary material for: A Light-Up Probe for Detection of Adenosine in Urine Samples by a Combination of an AIE Molecule and an Aptamer
Source: Sensors (Basel). 2017 Sep 29;17(10):2246. doi: 10.3390/s17102246 (PMC5677307; doi:10.3390/s17102246)
Supplement: Supplementary file 1 [file sensors-17-02246-s001.pdf]

Article

A light-up probe for detection of adenosine in urine samples by a combination of an AIE molecule and an aptamer

Ying-Ying Hu [1‡], Jing-Jing Liu[1‡], Xiang-Yu You [1], Can Wang [2], Zhen Li [2] and Wei-Hong Xie [1*]

[1] Department of Food and Pharmaceutical Engineering, Key Laboratory of Fermentation Engineering (Ministry of Education), Hubei University of Technology, Wuhan 430068, China. 1289642500@qq.com (Y.Y.H.); 1581226846@qq.com (J.J.L.); limnamil@163.com (X.Y.Y.); weihong.xie@mail.hbut.edu.cn (W.H.X.).

[2] Department of Chemistry, Wuhan University, Wuhan 430072, China; canwang-scola@whu.edu.cn (C.W.); lizhen@whu.edu.cn (Z.L.).

‡ Ying-Ying Hu and Jing-Jing Liu contributed equally to this work.

*Correspondence: weihong.xie@mail.hbut.edu.cn; Tel.: +86-139-7113-5198

Table S1 The recipe of different concentrations of the TPE-2N+ solutions for the examination of the emission behavior of TPE fluorogen in tris buffer

Sample TPE-2N+ (μM)	100 μM TPE-2N+ (μL)	Tris (μL)
**0**	0	200
**5**	10	190
**10**	20	180
**20**	40	160
**50**	100	100
**100**	200	0

Table S2 The recipe for the investigation of the viability of (TPE-2N+) + ABA probe

curve	Tris- HCl (μL)	100 μM TPE (μL)	100 μM ABA (μL)	1 μM Adenosine (μL)
**a**	200	0	0	0
**b**	180	20	0	0
**c**	180	20	0.2	0
**d**	160	20	0	20
**e**	160	20	0.2	20

Table S3 The recipe of different concentrations of nucleotide samples the specificity experiment

Sample nucleotides (μM)	100 μM TPE (μL)	100 μM ABA (μL)	0.001 μM nucleotides (μL)	10 mM Tris (μL)
**0**	20	0.2	0	180
**0.00001**	20	0.2	2	178
**0.00005**	20	0.2	10	170
**0.0001**	20	0.2	20	160
**0.0005**	20	0.2	100	80
			**1 μM nucleotide (μL)**	
**0.001**	20	0.2	0.2	180
**0.005**	20	0.2	1	179
**0.01**	20	0.2	2	178
**0.05**	20	0.2	10	170
**0.1**	20	0.2	20	160

Table S4 The intra-day precision of the detection by the probe for urine samples

Adenosine Added (μM)	sample1 Found (μM)	sample2 Found (μM)	sample3 Found (μM)	RSD (%, n=3)
0.01	0.0107	0.0024	0.0147	2.0
0.05	0.0472	0.0371	0.0448	1.5
0.1	0.0762	0.0885	0.0933	2.3

Table S5 The inter-day precision of the detection by the probe for urine samples

Adenosine Added (μM)	sample1 Found (μM)	sample2 Found (μM)	sample3 Found (μM)	RSD (%, n=3)
0.01	0.0069	0.0160	0.0036	2.1
0.05	0.0434	0.0481	0.0399	1.2
0.1	0.0986	0.0870	0.0916	1.5
